# Impact of P2Y12 inhibitors on clinical outcomes in sepsis-3 patients receiving aspirin: a propensity score matched analysis

**DOI:** 10.1186/s12879-024-09421-x

**Published:** 2024-06-11

**Authors:** Shaojun Jiang, Jianwen Xu, Chengjie Ke, Pinfang Huang

**Affiliations:** 1 Department of Pharmacy, The First Affiliated Hospital of Fujian Medical University, Fuzhou, China; 2 Department of Pharmacy, National Regional Medical Center, Binhai Campus of the First Affiliated Hospital, Fujian Medical University, Fuzhou, China

**Keywords:** Sepsis, P2Y12 inhibitors, Inflammation, Thrombosis, Bleeding

## Abstract

**Background:**

Sepsis is a life-threatening disease accompanied by disorders of the coagulation and immune systems. P2Y12 inhibitors, widely used for arterial thrombosis prevention and treatment, possess recently discovered anti-inflammatory properties, raising potential for improved sepsis prognosis.

**Method:**

We conducted a retrospective analysis using the data from Medical Information Mart for Intensive Care-IV database. Patients were divided into an aspirin-alone group versus a combination group based on the use of a P2Y12 inhibitor or not. Differences in 30-day mortality, length of stay (LOS) in intensive care unit (ICU), LOS in hospital, bleeding events and thrombotic events were compared between the two groups.

**Result:**

A total of 1701 pairs of matched patients were obtained by propensity score matching. We found that no statistically significant difference in 30-day mortality in aspirin-alone group and combination group (15.3% vs. 13.7%, log-rank *p* = 0.154). In addition, patients received P2Y12 inhibitors had a higher incidence of gastrointestinal bleeding (0.5% vs. 1.6%, *p* = 0.004) and ischemic stroke (1.7% vs. 2.9%, *p* = 0.023), despite having a shorter LOS in hospital (11.1 vs. 10.3, days, *p* = 0.043). Cox regression showed that P2Y12 inhibitor was not associated with 30-day mortality (HR = 1.14, 95% CI 0.95–1.36, *p* = 0.154).

**Conclusion:**

P2Y12 inhibitors did not provide a survival benefit for patients with sepsis 3 and even led to additional adverse clinical outcomes.

**Supplementary Information:**

The online version contains supplementary material available at 10.1186/s12879-024-09421-x.

## Introduction

Sepsis is a life-threatening organ dysfunction caused by a dysregulated host response to infection and is accompanied by multisystem and multi-organ dysfunction [1]. Despite advances in critical care medicine and antimicrobial therapy, patients with sepsis still face a high risk of death, with an average 30-day mortality rate of 24.4% [2]. The pathophysiology of sepsis is complex and multifaceted, including excessive and uncontrolled inflammatory responses and coagulation disorders [3]. These changes are interconnected and influence each other in a vicious cycle during sepsis. At present, the management of sepsis is mainly focused on infection control and symptomatic supportive care, and precision immunotherapy is still in the exploratory stage [4]. The lack of specific drugs targeting its pathophysiologic processes highlights the need for new therapeutic strategies.

P2Y12 inhibitors were initially used as antiplatelet agents for the prevention and treatment of myocardial infarction and ischemic stroke, with clopidogrel and ticagrelor commonly used. Emerging evidences suggest that P2Y12 inhibitors can improve tissue ischemia uncontrolled immune response in sepsis by inhibiting platelet activation and aggregation, modulating the immune response, and regulating the movement of leukocytes [5, 6]. In addition, P2Y12 inhibitors are able to counteract platelet-driven immune responses and inhibit monocyte and macrophage functions directly [7, 8]. Ticagrelor also attenuates inflammation by inhibiting adenosine uptake [9]. Relevant studies have demonstrated that P2Y12 inhibitors reduce the incidence of infectious diseases [10, 11] and reduce the burden of bronchial inflammation [12]. The discovery of this inflammatory modulating effect makes it promising as a potential treatment for infectious diseases.

As far as the pharmacodynamic mechanism is concerned, P2Y12 inhibitors fit the pathophysiologic changes of sepsis and have the probability of improving the prognosis. researchers are also optimistic about the application of P2Y12 inhibitors in sepsis [7]. However, the efficacy of P2Y12 in sepsis remains unclear. Considering the inevitable risk of bleeding from P2Y12 inhibitors, the trade-off between safety and efficacy still needs to be further explored. This study aims to evaluate the feasibility of P2Y12 inhibitors as an adjunctive treatment option for sepsis. In this study, we evaluated the effect of P2Y12 inhibitors on survival status, length of stay (LOS) in hospital and LOS in ICU in patients with sepsis. In addition, we also assessed the safety and efficacy of P2Y12 inhibitors in septic patients by comparing the incidence of bleeding events and thrombotic events separately.

## Method

### Data source

This retrospective analysis utilized data from the Medical Information Mart for Intensive Care IV (MIMIC-IV) database version 2.2 [13], which contains information on 299,712 patients who were admitted to Beth Israel Deaconess Medical Center from 2008 to 2019. One of the authors, SJ, has passed the online training in the Collaborative Institutional Training Initiative program and was granted access to the database (certification number: 59841319). Given the anonymized nature of the data, ethical approval was waived by the Ethics Committee of the First Affiliated Hospital of Fujian Medical University.

### Study population

Since the P2Y12 inhibitor is rarely administered alone, the target population in this study was limited to septic patients using aspirin. The diagnosis of sepsis is based on the sepsis-3 criteria proposed in 2016 [1]. After that, the patients were divided into aspirin-alone group or combination group according to whether they received P2Y12 inhibitors. P2Y12 inhibitors considered in this study included clopidogrel, prasugrel, ticagrelor and cangrelor. For patients with multiple hospitalizations or ICU admissions records, each hospitalization was considered as a separate sample, but only the first ICU record from each hospitalization was included. The specific inclusion criteria are as follows:

Inclusion criteria: (1) meeting the criteria of sepsis-3; (2) treatment with aspirin prior to the onset of sepsis.

Exclusion criteria: (1) age < 18 years; (2) LOS in ICU less than 48 h; (3) duration of aspirin and/or P2Y12 inhibitors less than 3 days; (4) received two or more P2Y12 inhibitors during hospitalization; (5) more than 30% of missing data at baseline or missing clinical outcomes.

### Data extraction

We extracted the following information: (1) Demographic information: including age, gender, race, weight, height, smoking, alcohol use. (2) Highest sequential organ failure assessment (SOFA) score during hospitalization, primary site of infection, comorbidities and therapy received during hospitalization. Comorbidities include hypertension, diabetes, neoplasms, chronic obstructive pulmonary disease (COPD), history of coronary artery surgery (CAS) and history of thrombosis. Therapy received during hospitalization include duration of heparin, oral anticoagulants, statins, immunomodulators, mechanical ventilation, continuous renal replacement therapy (CRRT). Oral anticoagulants include warfarin, apixaban, rivaroxaban, edoxaban, and dabigatran. Immunomodulators include mycophenolate, tacrolimus, cyclosporine, leflunomide, and hydroxychloroquine. (3) First laboratory results after admission, including international normalized ratio (INR) and prothrombin time (PT).

### Outcomes

Primary outcomes were 30-day mortality rate, bleeding events and thrombotic events. Bleeding events were considered as safety indicators, including gastrointestinal bleeding (GIB) and intracranial hemorrhage (ICH). Thrombotic events were considered as efficacy indicators, including venous thromboembolism (VTE) and ischemic stroke, the former including deep vein thrombosis and pulmonary embolism. Clinical events were collected from admission to discharge and identified by international classification of diseases (ICD) codes. Secondary outcome included LOS in hospital and LOS in ICU.

### Statistical analysis

Missing data were treated with simple interpolation or multiple interpolation depending on their proportion. The specific method for each variable was shown in the Appendix. Propensity score matching (PSM) was used to narrow the baseline difference between the two groups. The propensity score was calculated by logistic regression, based on demographic characteristics, SOFA score, primary location of infection, comorbidities, therapy and laboratory results. The caliper value was set to 0.05.

After using the Anderson-Darling test and Levene’s Test to verify normality and variance homogeneity of continuous variables, respectively, the results showed that none of the continuous variables satisfied either normality or variance chi-square at the same time. Therefore, continuous variables were presented as median (quartile) and compared using paired Wilcoxon tests. Categorical variables were presented as frequency (percentage) and compared using the chi-square test or Fisher’s exact test. The 30-day survival curve was generated using the Kaplan-Meier method and compared by log-rank test. Univariate Cox regression was used to initially analyze baseline information related to 30-day mortality. After that, characteristics with a p value of less than 0.10 were entered into a multivariate Cox regression. All statistical analyses were done using R software 4.2.0 (R Core Team, 2023). P values were reported as two-sided. Statistical differences were set as p-values less than or equal to 0.05.

## Result

### Baseline

**Fig. 1:**
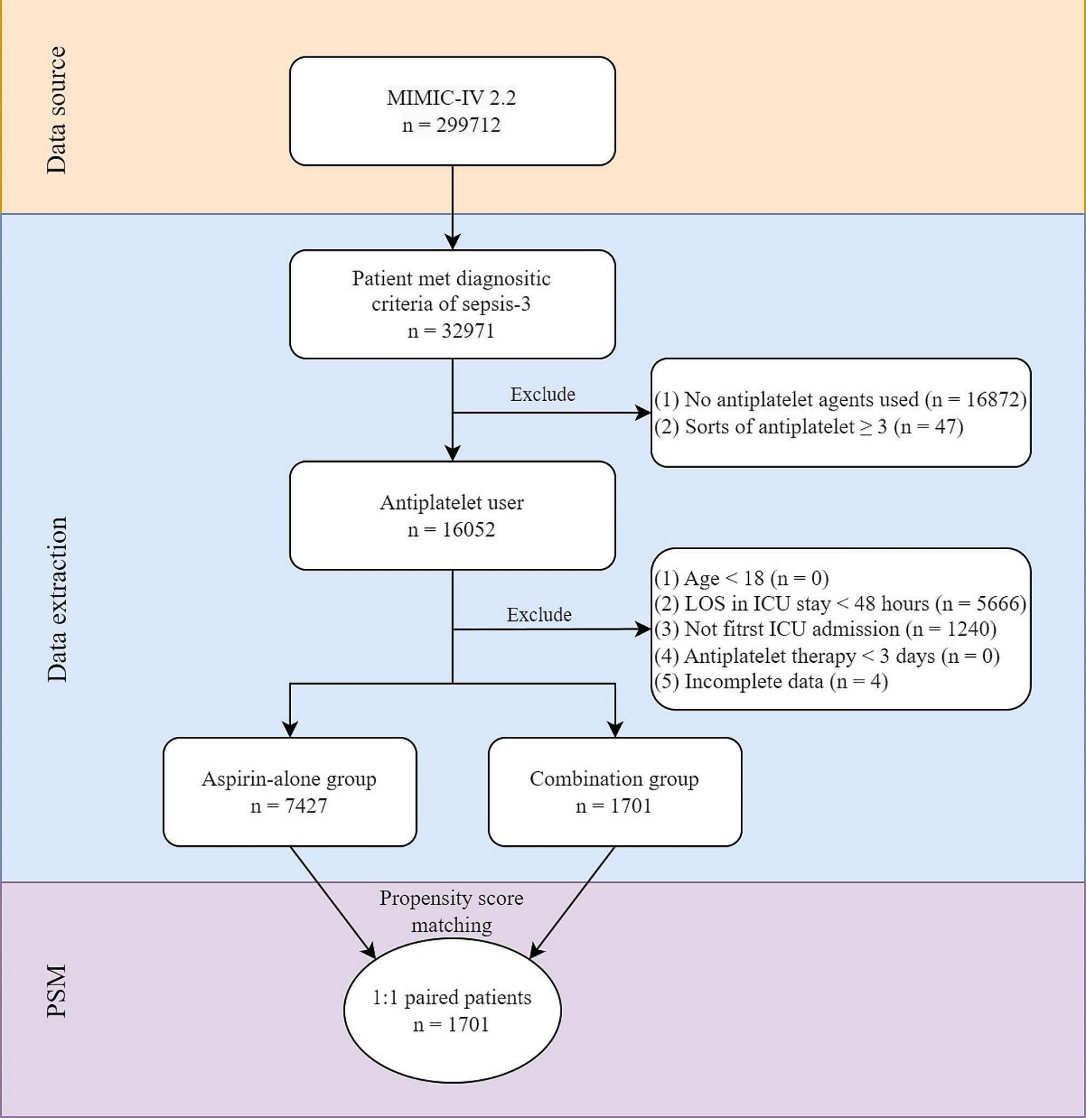
Flow chart of patient screening

The patient screening process is shown in Fig. 1. A total of 16,052 sepsis patients using aspirin were preliminarily identified from the MIMIC-IV database according to the sepsis-3 criteria. After further screening 7458 cases were included in the aspirin alone group and 1684 cases in the combination group, respectively. Subsequently, 1701 pairs 1:1 matched patient were obtained by PSM. In the matched cohort, the combination group included 1597 patients with clopidogrel, 31 patients with prasugrel and 73 patients with ticagrelor. None of the patients were treated with cangrelor.

The baseline of patients after PSM is shown in Table 1. Except for the first INR, the rest of the characteristics baseline were not statistically different between the two groups (*p* > 0.05). Overall, the elderly was the main group in this study, with a median age of approximately 72 years. Males accounted for more than 60% in both groups. In terms of race, whites had the highest percentage about 80%. Blacks were the next highest, more than 8%. The rest of the race groups had percentages of less than 5%. Hypertension was the most common comorbidity, observed in more than 80% of patients. In addition, nearly half of the patients had comorbid diabetic. There was also no significant difference in the duration of heparin therapy prior to the sepsis between the two groups (9, 4–15 vs. 8,4–14 days, *p* = 0.259). Baseline information before PSM in both groups can be found in the Appendix (Table S1).

**Table 1:** Baseline characteristics in two groups after propensity score matching

Characteristic	Aspirin-alone group *N* = 1701	Combination group *N* = 1701	*P* value
Age	71.9 (63.7, 80.5)	71.6 (62.6, 81.0)	0.666
Gender, n (%)			0.861
Male	1030 (60.6%)	1036 (60.9%)	
Female	671 (39.4%)	665 (39.1%)	
Race, n (%)			0.679
White	1346 (79.1%)	1373 (80.7%)	
Black	154 (9.1%)	139 (8.2%)	
Asian	49 (2.9%)	50 (2.9%)	
Hispanic	67 (3.9%)	68 (4.0%)	
Others	85 (5.0%)	71 (4.2%)	
Weight/kg	80.0 (68.2, 93.5)	80.0 (67.6, 94.9)	0.988
Height/cm	170.0 (163.0, 178.0)	170.0 (160.0, 178.0)	0.935
Smoke, n (%)	360 (21.2%)	356 (20.9%)	0.900
Alcohol use disorder, n (%)	64 (3.8%)	62 (3.6%)	0.928
SOFA score	7.0 (5.0, 10.0)	7.0 (5.0, 10.0)	0.757
Primary location of infection, n (%)			0.918
Respiratory	449 (26.4%)	423 (24.9%)	
Genitourinary	232 (13.6%)	231 (13.6%)	
Skin or subcutaneous tissue	55 (3.2%)	65 (3.8%)	
Gastrointestinal	47 (2.8%)	48 (2.8%)	
Implant	31 (1.8%)	33 (1.9%)	
Abdomen	50 (2.9%)	40 (2.4%)	
Endocarditis	17 (1.0%)	16 (0.9%)	
Bloodstream	7 (0.4%)	8 (0.5%)	
Other / Unspecified	813 (47.8%)	837 (49.2%)	
Comorbidities, n (%)			
Hypertension	1368 (80.4%)	1362 (80.1%)	0.830
Diabetes	859 (50.5%)	855 (50.3%)	0.918
Neoplasm	363 (21.3%)	356 (20.9%)	0.801
COPD	150 (8.8%)	148 (8.7%)	0.952
History of CAS	682 (40.1%)	692 (40.7%)	0.753
History of thrombosis	309 (18.2%)	303 (17.8%)	0.823
Therapy, n (%)			
Duration of heparin, day	9.0 (4.0, 15.0)	8.0 (4.0, 14.0)	0.563
Oral anticoagulants	494 (29.0%)	470 (27.6%)	0.382
Statins	1461 (85.9%)	1450 (85.2%)	0.626
Immunomodulators	120 (7.1%)	115 (6.8%)	0.787
Mechanical ventilation	1125 (66.1%)	1129 (66.4%)	0.913
CRRT	193 (11.3%)	189 (11.1%)	0.871
INR	1.2 (1.1, 1.5)	1.2 (1.1, 1.4)	0.018
PT, second	13.7 (12.3, 16.3)	13.6 (12.2, 15.8)	0.163

*Note* SOFA, sequential organ failure assessment; COPD, chronic obstructive pulmonary disease; CAS, coronary artery surgery; CRRT, continuous renal replacement therapy; INR, international normalized ratio; PT, prothrombin time

### Clinical outcomes

Clinical outcomes for both groups are summarized in Table 2. Patients in the aspirin-alone group had longer LOS in hospital compared to the combination group (11.1,6.9–18.7 vs. 10.3, 6.9–17.2, days, *p* = 0.043). We performed an additional analysis of survival patients and similarly observed longer LOS in hospital in the aspirin-alone group (12.1, 7.4–20.5 vs. 10.8, 6.7–17.7, days, *p* = 0.028). No statistical difference was found in LOS in ICU between the two groups (4.3, 2.9–8.6 vs. 4.7, 3.1–7.8, days, *p* = 0.760). The combination group had a higher incidence of GIB (0.5% vs. 1.6%, *p* = 0.004) and ischemic stroke (1.7% vs. 2.9%, *p* = 0.023). ICH (1.6% vs. 1.5%, *p* = 0.782) and VTE (5.4% vs. 4.9%, *p* = 0.535) occurred at similar rates in both groups and there was no statistical difference.

**Table 2:** Clinical outcomes among aspirin-alone group and combination group

Outcomes	Aspirin-alone group *N* = 1701	Combination group *N* = 1701	*P* value
LOS in hospital, day	11.1 (6.9, 18.7)	10.3 (6.9, 17.2)	0.043
LOS in ICU, day	4.3 (2.9, 8.6)	4.7 (3.1, 7.8)	0.760
GIB, n (%)	9 (0.5%)	27 (1.6%)	0.004
ICH, n (%)	28 (1.6%)	25 (1.5%)	0.782
VTE, n (%)	92 (5.4%)	83 (4.9%)	0.535
Ischemic stroke, n (%)	29 (1.7%)	50 (2.9%)	0.023

*Note* LOS, length of stay; GIB, gastrointestinal bleeding; ICH, intracerebral hemorrhage; VTE, venous thromboembolism

### Survival analysis

**Fig. 2:**
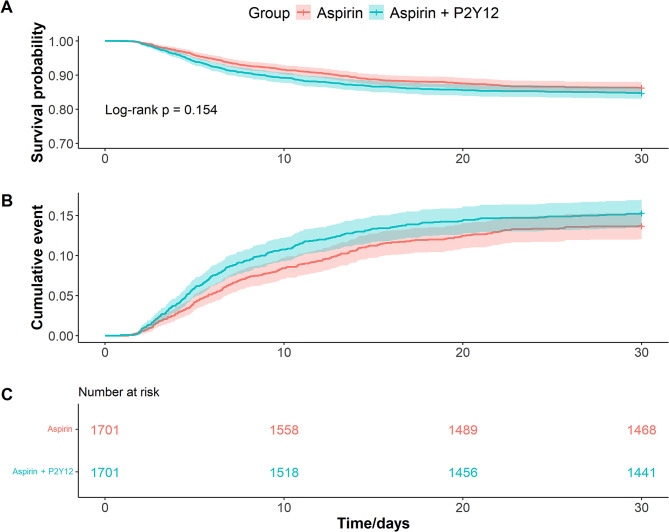
Kaplan-Meier curves for 30-day mortality in matched data. Note: **A**, survival probability of patients in the aspirin-alone and combination groups over time. **B**, cumulative risk of 30-day mortality. **C**, the number of patients at risk at each time point

Figure 2 shows the Kaplan-Meier curves for 30-day survival in both groups. Survival analysis showed that the 30-day survival status was similar in both groups (86.3% vs. 84.7%, log-rank test: p-value = 0.154).

**Fig. 3:**
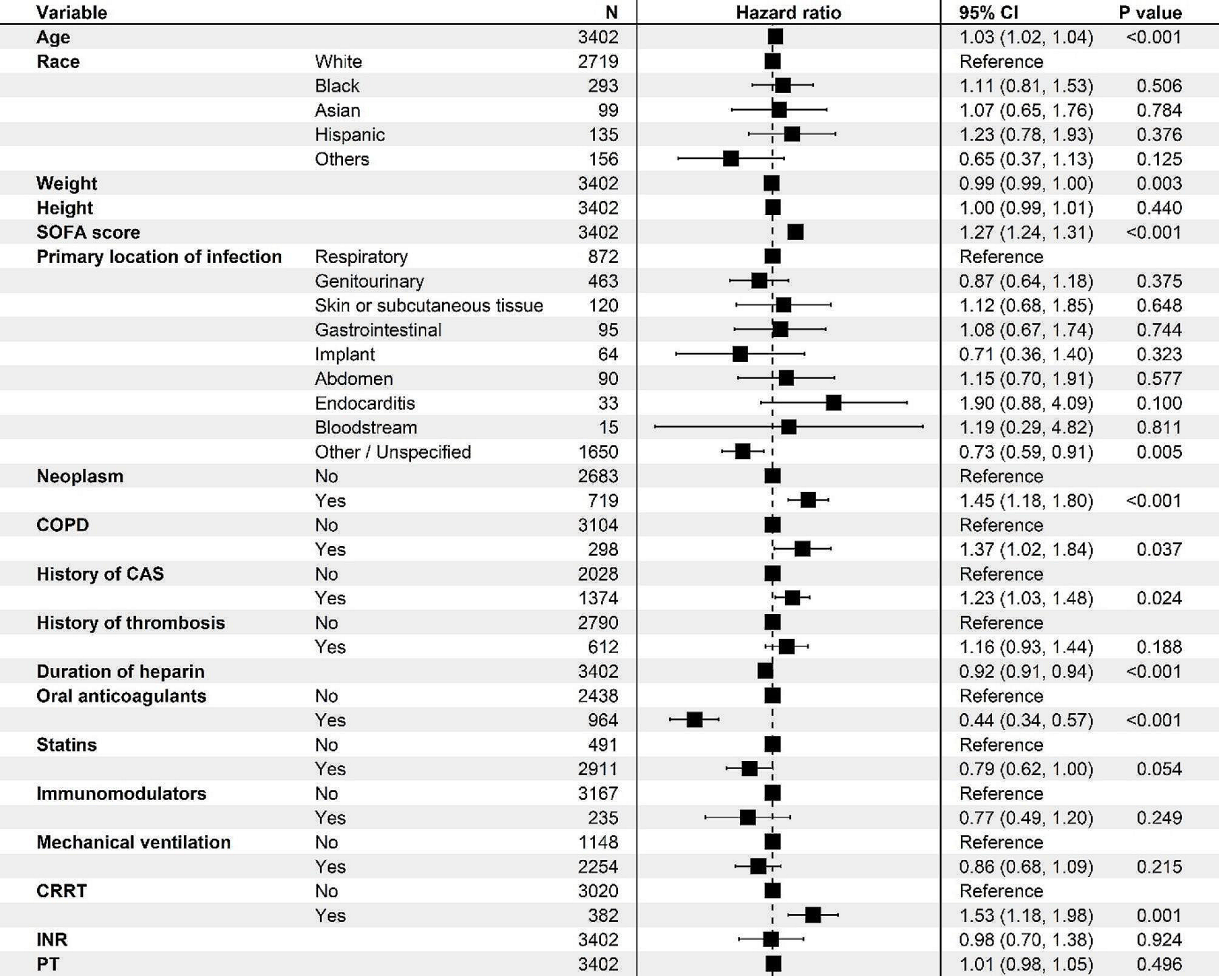
Forest plot of multivariate cox regression for 30-day mortality after propensity score matching. Note: SOFA: sequential organ failure assessment; COPD: chronic obstructive pulmonary disease; INR: international normalized ratio; PT, prothrombin time; HR, hazard ratio; CI, confidence interval

In univariate Cox regression, age, race, weight, height, SOFA score, primary location of infection, neoplasm, COPD, history of CAS, history of thrombosis, duration of heparin, anticoagulants, statin, immunomodulator, mechanical ventilation, CRRT, INR and PT correlated with 30-day mortality (*p* < 0.10). The results of the univariate Cox regression are supplemented in the Appendix Table S2. P2Y12 inhibitors were not associated with 30-day mortality and were excluded from entering multivariate Cox regression (HR = 1.14, 95% CI 0.95–1.36, *p* = 0.154). The results of the multivariate Cox regression of the above 18 variables with 30-day mortality are presented as a forest plot (Fig. 3). The multivariate Cox regression analyses demonstrated that the effects of age (HR = 1.03, 95% CI 1.02–1.04, *p* < 0.001), SOFA score (HR = 1.27, 95% CI 1.24–1.31, *p* < 0.001), neoplasm (HR = 1.45, 95% CI 1.18–1.80, *p* = 0.001), COPD (HR = 1.37, 95% CI 1.02–1.84, *p* = 0.037), history of CAS (HR = 1.23, 95% CI 1.03–1.48, *p* = 0.024) and CRRT (HR = 1.53, 95% CI 1.18–1.98, *p* = 0.024) were the risk factors for 30-day mortality.

In contrast, a total of four protective factors associated with 30-day mortality were observed. For every 1 kg increase in weight, the risk of 30-day mortality decreases by 1% (HR = 0.99, 95% CI 0.99-1.00, *p* = 0.003). Patients without specific infections demonstrated a lower risk of 30-day mortality (HR = 0.73, 95% CI 0.59–0.91, *p* = 0.005). For each additional day of heparin duration, the risk of 30-day mortality decreased by 8% (HR = 0.92, 95% CI 0.91–0.94, *p* < 0.001). Oral anticoagulants had a significant and negative effect on 30-day mortality (HR = 0.44, 95% CI 0.34–0.57, *p* < 0.001).

## Discussion

In this study, we conducted a retrospective study based on data from the MIMIC-IV database to compare differences in clinical outcomes among sepsis patients treated with aspirin or aspirin combined with P2Y12 inhibitors. The main finding of this study was that P2Y12 inhibitors did not improve 30-day mortality in patients with sepsis, despite shortening the LOS in hospital. In contrast, P2Y12 inhibitors brought to a higher risk of GIB. Surprisingly, ischemic stroke was more prevalent in the combination group compared with the aspirin-alone group. This difference may be due to grouping, i.e., patients on P2Y12 inhibitors naturally had a higher risk of ischemic stroke, even after PSM.

In the available studies, early evidence demonstrates that P2Y12 inhibitors can play a positive role in the prevention or treatment of sepsis [14–16]. However, after the definition of sepsis was updated to sepsis-3 in 2016 [1], the above findings may not apply to the latest sepsis criteria. Among other infections not defined as sepsis-3, antibiotic-like effects of P2Y12 inhibitors have been reported on infections caused by Staphylococcus aureus [17]. P2Y12 inhibitors could reduce the incidence and in-hospital mortality of Staphylococcus aureus bacteremia [11, 18]. Unlike bacterial infections, P2Y12 inhibitors appear to be ineffective against infections with viruses [19]. Under the sepsis-3 criterion, to the best of our knowledge, the present study is the first to assess the impact of P2Y12 inhibitors on clinical outcomes. However, this study was limited by the sample size, with only 73 patients on ticagrelor and 133 patients cultured for Staphylococcus aureus, so subgroup analyses were not performed.

Among the impacting factors associated with 30-day mortality revealed by multivariate Cox regression, longer heparin duration and oral anticoagulants made patients more likely to survive post-sepsis. In addition, statins may also have a potential protective effect (*p* = 0.054 in the PSM cohort and *p* < 0.001 in total cohort). These protective factors are consistent with previous literature [20–22]. These therapeutic measures are expected to reduce mortality in sepsis patients if applied in a timely manner in the clinical setting. Currently, machine learning methods based on admission data or real-time data have been able to predict the risk of sepsis during hospitalization with a high degree of accuracy [23, 24]. Therefore, early intervention in patients before the onset of sepsis is entirely possible. However, clinical studies are still needed to assess the clinical benefit of early intervention. In addition, older age, high SOFA score, neoplasm, COPD, history of CAS and CRRT were identified as risk factors for 30-day mortality, consistent with findings from prior studies [25–27]. It is necessary to provide additional medical attention to this group of patients.

Overall, the use of P2Y12 inhibitors did not improve sepsis outcomes and even had the opposite effect. Whether the specific mechanism is related to the anti-inflammatory effect still requires further study. The role of cangrelor in sepsis has not been fully investigated. In addition, some researchers have appealed for the development of drugs derived from ticagrelor with no antiplatelet activity to avoid the risk of bleeding and thus achieve higher administration doses [28]. Considering the lack of high-strength clinical studies and the inconsistency of conclusions, P2Y12 inhibitors are not currently recommended as adjunctive therapy to ameliorate inflammation and coagulation disorders in sepsis.

There are several important considerations to note regarding the limitations of this study. Firstly, the reliability of the study heavily relies on the quality and accuracy of the data contained within the MIMIC-IV database. Since this study is retrospective in nature, it is susceptible to inherent limitations such as selection bias and confounding variables that were not measured or accounted for. The retrospective attributes undermine the strength of causal conclusions drawn from the analysis. Secondly, the method of identifying clinical outcomes through ICD codes may introduce a risk of overdiagnosis. This limitation is supposed to bring about the same impact between the two groups. Therefore, it does not lead to a change in statistical differences. Furthermore, it’s important to recognize that the burden of sepsis is closely linked to the level of social development. Since the MIMIC-IV data originates from the United States, a high-income country with a well-established healthcare system, caution must be exercised when attempting to generalize these findings to populations in resource-limited settings.

## Conclusion

Although P2Y12 inhibitors reduce the incidence of VTE, they do not improve 30-day mortality and increase the risk of GIB. Patients with sepsis have a low likelihood of benefiting from P2Y12 inhibitor.

### Electronic supplementary material

Below is the link to the electronic supplementary material.

Supplementary Material 1

## Data Availability

The original data were obtained from https://physionet.org/content/mimiciv/2.2/. The SQL script for preliminary data extraction was obtained from https://github.com/MIT-LCP/mimic-code. The R code used for data organization and statistics was released at https://github.com/simpleseasalt/sepsis_P2Y12.

## Abbreviations

CAS: Coronary Artery Surgery
COPD: Chronic Obstructive Pulmonary Disease
GIB: Gastrointestinal Bleeding
HR: Hazard Ratio
ICD: International Classification of Diseases
ICU: Intensive Care Unit
ICH: Intracerebral Hemorrhage
INR: International Normalized Ratio
MIMIC-IV: Medical Information Mart for Intensive Care IV
LOS: Length Of Stay
PSM: Propensity Score Matching
PT: Prothrombin Time
SOFA: Sequential Organ Failure Assessment
VTE: Venous Thromboembolism
