# Supplementary material for: Impact of P2Y12 inhibitors on clinical outcomes in sepsis-3 patients receiving aspirin: a propensity score matched analysis
Source: BMC Infect Dis. 2024 Jun 11;24:575. doi: 10.1186/s12879-024-09421-x (PMC11167871; doi:10.1186/s12879-024-09421-x)
Supplement: Supplementary file 1 — Supplementary Material 1 [file 12879_2024_9421_MOESM1_ESM.docx]

Imputation of missing value

Missing data at baseline are shown in Figure S1. There were no missing data on age, ethnicity, duration of heparin, and comorbidities. The missing data for weight, INR, and PT were less than 10%, so median interpolation was used. The missing data for height were more than 10% but less than 40%. And Little's test showed the missing data were not completely randomly distributed (p < 0.05). Random forest-based multiple interpolation was used to predict and complement the data with mice package.


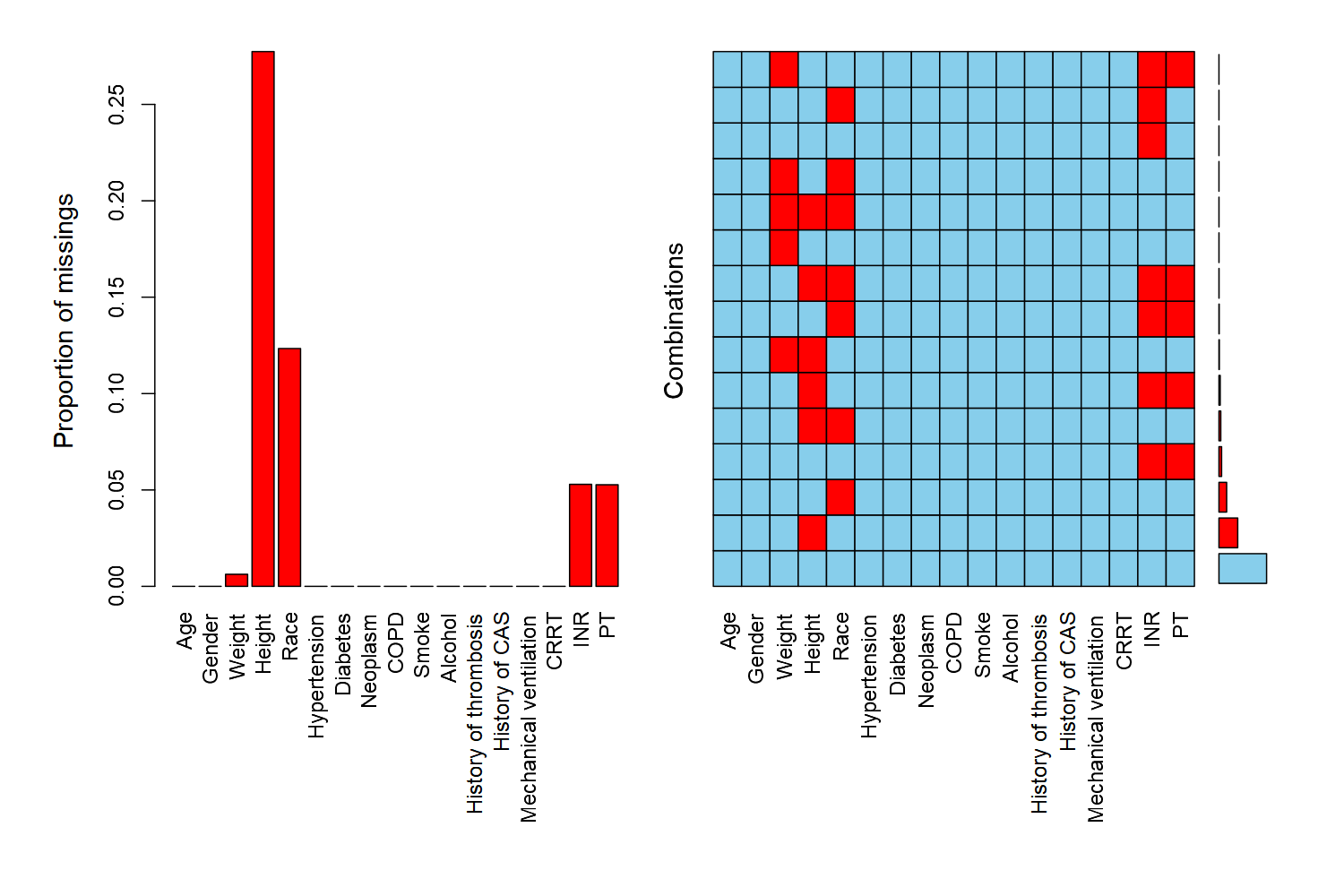


Figure S1: Missing Value of Baseline Characteristics.

Note: Missing value is indicated in red. COPD: chronic obstructive pulmonary disease; CAS, coronary artery surgery; INR: international normalized ratio; PT, prothrombin time.

Table S1: Baseline Characteristics in Two Groups before Propensity Score Matching

| Characteristic | Aspirin-alone group  N = 7427 | Combination group  N = 1701 | P value |
| --- | --- | --- | --- |
| Age | 71.7 (62.4, 80.6) | 71.6 (62.6, 81.0) | 0.504 |
| Gender, n (%) |  |  | 0.276 |
| Male | 4414 (59.4%) | 1036 (60.9%) |  |
| Female | 3013 (40.6%) | 665 (39.1%) |  |
| Race, n (%) |  |  | 0.008 |
| White | 5897 (79.4%) | 1373 (80.7%) |  |
| Black | 814 (11.0%) | 139 (8.2%) |  |
| Asian | 194 (2.6%) | 50 (2.9%) |  |
| Hispanic | 253 (3.4%) | 68 (4.0%) |  |
| Others | 269 (3.6%) | 71 (4.2%) |  |
| Weight/kg | 80.3 (68.1, 96.0) | 80.0 (67.6, 94.9) | 0.076 |
| Height/cm | 170.0 (163.0, 178.0) | 170.0 (160.0, 178.0) | 0.621 |
| Smoke, n (%) | 1466 (19.7%) | 356 (20.9%) | 0.283 |
| Alcohol use disorder, n (%) | 321 (4.3%) | 62 (3.6%) | 0.234 |
| SOFA score | 7.0 (5.0, 9.0) | 7.0 (5.0, 10.0) | < 0.001 |
| Primary location of infection, n (%) |  |  | 0.318 |
| Respiratory | 1870 (25.2%) | 423 (24.9%) |  |
| Genitourinary | 915 (12.3%) | 231 (13.6%) |  |
| Skin or subcutaneous tissue | 260 (3.5%) | 65 (3.8%) |  |
| Gastrointestinal | 244 (3.3%) | 48 (2.8%) |  |
| Implant | 176 (2.4%) | 33 (1.9%) |  |
| Abdomen | 154 (2.1%) | 40 (2.4%) |  |
| Endocarditis | 113 (1.5%) | 16 (0.9%) |  |
| Bloodstream | 53 (0.7%) | 8 (0.5%) |  |
| Other / Unspecified | 3642 (49.0%) | 837 (49.2%) |  |
| Comorbidities, n (%) |  |  |  |
| Hypertension | 5627 (75.8%) | 1362 (80.1%) | < 0.001 |
| Diabetes | 2932 (39.5%) | 855 (50.3%) | < 0.001 |
| Neoplasm | 1820 (24.5%) | 356 (20.9%) | 0.002 |
| COPD | 504 (6.8%) | 148 (8.7%) | 0.007 |
| History of CAS | 1460 (19.7%) | 692 (40.7%) | < 0.001 |
| History of thrombosis | 1042 (14.0%) | 303 (17.8%) | < 0.001 |
| Therapy, n (%) |  |  |  |
| Duration of heparin, day | 7.0 (2.0, 14.0) | 8.0 (4.0, 14.0) | < 0.001 |
| Oral anticoagulants | 2547 (34.3%) | 470 (27.6%) | < 0.001 |
| Statins | 4960 (66.8%) | 1450 (85.2%) | < 0.001 |
| Immunomodulators | 300 (4.0%) | 115 (6.8%) | < 0.001 |
| Mechanical ventilation | 5180 (69.7%) | 1129 (66.4%) | 0.007 |
| CRRT | 570 (7.7%) | 189 (11.1%) | < 0.001 |
| INR | 1.3 (1.1, 1.5) | 1.2 (1.1, 1.4) | < 0.001 |
| PT, second | 14.1 (12.5, 16.7) | 13.6 (12.2, 15.8) | < 0.001 |

Note: SOFA, sequential organ failure assessment; COPD, chronic obstructive pulmonary disease; CAS, coronary artery surgery; CRRT, continuous renal replacement therapy; INR, international normalized ratio; PT, prothrombin time.


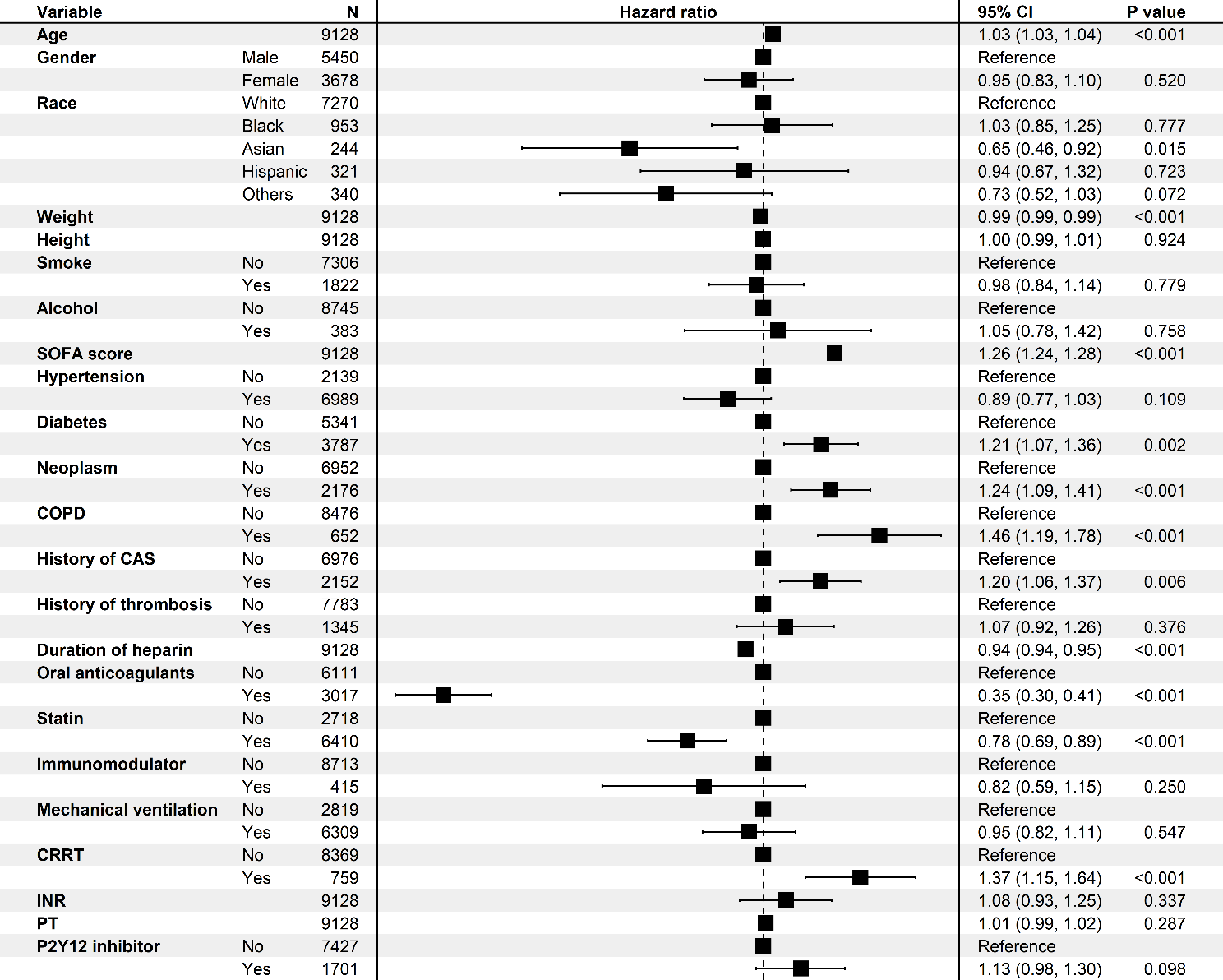


Figure S2: Forest Plot of Multivariate Cox Regression before Propensity Score Matching

Note: SOFA: sequential organ failure assessment; COPD: chronic obstructive pulmonary disease; INR: international normalized ratio; PT, prothrombin time.

Table S2: Results of Univariate Cox Regression After Propensity Score Matching.

| Characteristic | HR | Lower 95%CI | Upper 95%CI | P value |
| --- | --- | --- | --- | --- |
| Age | 1.03 | 1.02 | 1.04 | < 0.001 |
| Gender (Female) | 0.89 | 0.74 | 1.06 | 0.197 |
| Race |  |  |  |  |
| White | Reference |  |  |  |
| Black | 1.06 | 0.78 | 1.44 | 0.727 |
| Asian | 1.20 | 0.74 | 1.96 | 0.453 |
| Hispanic | 1.03 | 0.66 | 1.61 | 0.896 |
| Others | 0.60 | 0.35 | 1.02 | 0.058 |
| Weight | 0.99 | 0.99 | 1.00 | <0.001 |
| Height | 0.99 | 0.99 | 1.00 | 0.084 |
| Smoke | 0.85 | 0.68 | 1.07 | 0.167 |
| Alcohol | 0.75 | 0.44 | 1.28 | 0.298 |
| SOFA score | 1.22 | 1.20 | 1.25 | < 0.001 |
| Primary location of infection |  |  |  |  |
| Respiratory | Reference |  |  |  |
| Genitourinary | 0.72 | 0.54 | 0.98 | 0.034 |
| Skin or subcutaneous tissue | 0.88 | 0.54 | 1.43 | 0.600 |
| Gastrointestinal | 1.22 | 0.77 | 1.95 | 0.397 |
| Implant | 0.89 | 0.47 | 1.69 | 0.723 |
| Abdomen | 1.23 | 0.77 | 1.99 | 0.386 |
| Endocarditis | 1.23 | 0.58 | 2.63 | 0.589 |
| Bloodstream | 0.76 | 0.19 | 3.05 | 0.693 |
| Other / Unspecified | 0.71 | 0.58 | 0.88 | 0.002 |
| Hypertension | 0.95 | 0.76 | 1.18 | 0.629 |
| Diabetes | 1.10 | 0.92 | 1.31 | 0.305 |
| Neoplasm | 1.23 | 1.00 | 1.51 | 0.050 |
| COPD | 1.33 | 1.00 | 1.77 | 0.048 |
| History of CAS | 1.29 | 1.08 | 1.54 | 0.005 |
| History of thrombosis | 1.27 | 1.03 | 1.58 | 0.028 |
| Duration of heparin | 0.96 | 0.95 | 0.98 | < 0.001 |
| Oral anticoagulants | 0.46 | 0.36 | 0.58 | < 0.001 |
| Statins | 0.74 | 0.59 | 0.93 | 0.010 |
| Immunomodulators | 0.60 | 0.39 | 0.93 | 0.020 |
| Mechanical ventilation | 1.53 | 1.25 | 1.87 | < 0.001 |
| CRRT | 2.96 | 2.42 | 3.63 | < 0.001 |
| INR | 1.17 | 1.10 | 1.24 | < 0.001 |
| PT | 1.02 | 1.01 | 1.02 | < 0.001 |
| P2Y12 inhibitor | 1.14 | 0.95 | 1.36 | 0.154 |

Note: SOFA, sequential organ failure assessment; COPD, chronic obstructive pulmonary disease; CAS, coronary artery surgery; CRRT, continuous renal replacement therapy; INR, international normalized ratio; PT, prothrombin time. Variables with p-values less than 0.05 entered into multivariate Cox regression.
